# Identifying colon cancer stage related genes and their cellular pathways

**DOI:** 10.3389/fgene.2023.1120185

**Published:** 2023-01-19

**Authors:** Bolin Chen, Nandita Chakrobortty, Apu Kumar Saha, Xuequn Shang

**Affiliations:** ^1^ School of Computer Science, Northwestern Polytechnical University, Xi’an, Shaanxi, China; ^2^ MIIT Key Laboratory of Big Data Storage and Management, Northwestern Polytechnical University, Xi’an, Shaanxi, China; ^3^ National Engineering Laboratory for Integrated Aero-Space-Ground-Ocean Big Data Application Technology, Northwestern Polytechnical University, Xi’an, Shaanxi, China

**Keywords:** colon cancer, differentially expressed gene, cancer stage, functional evolution network, cancer evolution

## Abstract

In the world, colon cancer is regarded as one of the most common deadly cancer. Due to the lack of a better understanding of its prognosis system, this prevailing cancer has the second-highest morbidity and mortality rate compared with other cancers. A variety of genes are responsible to participate in colon cancer and the molecular mechanism is almost unsure. In addition, various studies have been done to identify the differentially expressed genes to investigate the dysfunctions of the genes but most of them did it individually. In this study, we constructed a functional interaction network for identifying the group of genes that conduct cellular functions and Protein-Protein Interaction network, which aims to better understanding protein functions and their biological relationships. A functional evolution network was also generated to analyze the dysfunctions from initial stage to later stage of colon cancer by investigating the gene modules and their molecular functions. The results show that the proposed evolution network is able to detect the significant cellular functions, which can be used to explore the evolution process of colon cancer. Moreover, a total of 10 core genes associated with colon cancer were identified, which were INS, SNAP25, GRIA2, SST, GCG, PVALB, SLC17A7, SLC32A1, SLC17A6, and NPY, respectively. The responsible candidate genes and corresponding pathways presented in this study could be used to develop new tumor indicators and novel therapeutic targets for the prevention and treatment of colon cancer.

## Introduction

Colon cancer is a cancer with high morbidity and mortality. It is considered as the most common malignant cancer and the second commonest death cause in the modern world. Genome instability, epigenetic abnormalities, and gene expression disorders are typical molecular features of colon cancer. The increase in prevalence is related to an aging population as well as poor eating habits, smoking, lack of physical movements, and obesity in western countries (Kuipers et al., 20162015). A change in incidence is also observed in some familial cancer syndromes as well as in sporadic disease rates. The incidence of the disease is more common in urban areas compared with rural areas. More men are injured by this disease rather than women. Although older people are at high risk, however, in recent years a significant number of young generations are also victims of this cancer (Chen et al., 2020a).

As living standards around the world have improved and access to healthcare has increased, we have noticed a considerable improvement in the diagnosis and treatment of diseases. Despite these medical advances, even though the death rate has reduced in over the world, however, the mortality rate from colon cancer has increased, and overall survival is still poor. Many analyses based on the survival rate have demonstrated that metastasis can play a vital role in the reduction of survival rate (Cho-Chung et al., 2002; He et al., 2018). However, previous studies did the analysis where DEGs act alone. But genes are not isolated from each other. They worked together by creating modules and forming modules to undertake biological functions (Chen et al., 2020b). In this study, we analyzed depending on the modules of genes. Moreover, various relevant pathways have already been discovered for colon cancer development but still there remain so many and the main reason is the complex evolutionary process of CRC development.

As colon cancer is considered the leading cause of death cancer in the world and the molecular mechanism of colon cancer is almost unclear. It has been widely accepted that the earlier stage of the cancer is significantly different than the later stages, and if a patient can be diagnosised earlier, it may have higher probability to be cured. In this study, we have performed several investigations based on both data and networks. Traditional approaches have limitations as follows. They did not employ modules thoroughly to evaluate the differentially expressed genes, but directly utilized DEGs to make the functional enrichment analyses. But it is known that proteins rarely act alone, but often collaborate in groups to carry out biological tasks. In this study we performed a cluster analysis on the functional interactional network to identify the modules of genes. These modules were used to construct the PPI network and the cluster interaction network. Moreover, to better understand the relationships between modules and examine their biological functions, we created an evolutionary functional network using the most important DEGs. In addition, existing models also used directly the expression data for their analysis to find out DEGs and enrichment analysis, whereas in this study we work with modules, we use the data from the functional interaction network, cluster interaction network, and PPI network to get the higher precision result.

**FIGURE 1:**
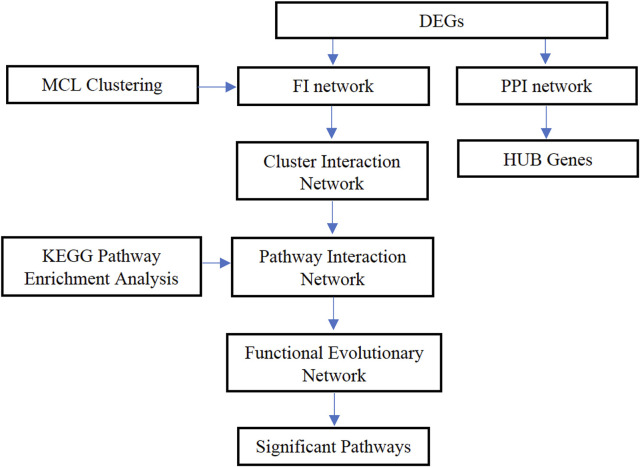
The overall framework of the proposed method.

To be more specific, data and network analysis were performed in different ways to identify the stage-related genes and understand the different biological functions of colon cancer. Gene expression data and colon cancer clinical information data were obtained from the TCGA database and divided the expression data of colon cancer samples in four stages. The differentially expressed genes (DEGs) were obtained at each pair by analyzing the gene expression data, and the PPI network and functional interaction network were constructed for analyzing the interaction among genes. Then, the MCL graph clustering algorithm was applied to select the modules of proteins. By combining the seven-cluster networks, a functional evolutionary network was finally established to analyze the relationship between functional modules for each stage and adjacent stages. The most relevant pathways were identified by doing a KEGG pathway enrichment analysis. In addition, the stage-related hub genes of colon cancer were also identified. This study is expected to provide some significant information regarding colon cancer at different stages and the potential biomarkers that can be used for early diagnosis and make a contribution to colon cancer treatment. The overall framework is shown in Figure 1.

## Materials and methods

### Gene expression data

Gene expression data were downloaded from The Cancer Genome Atlas (TCGA) for colon cancer. TCGA colon adenocarcinoma (TCGA-COAD) is the project name (https://portal.gdc.cancer.gov/projects/TCGA-COAD). HTSeq-Counts data is included in this database, along with HTSeq-FPKM and HT-FPKM-UQ data. The RNA-seq samples of data type HTSeq-Counts were analyzed for this analysis. The total number of samples was 329. And the total number of 20,530 protein-coding genes were selected for the next analysis.

### Clinical data and differentially expressed gene identification

Clinical data on colon cancer were also acquired from the TCGA dataset, which included 447 colon cancer samples. There was a variety of clinical information available in the original dataset for each sample, but for this study, only the sample number and four cancer stages information were retrieved here. After following the correlation of gene expression data with the corresponding clinical data and extracting the missing information data, got 312 overlapping samples. All these samples (overlapping sample numbers) were categorized into five groups. They are healthy human tissue or normal tissue, stage I, stage II, stage III, and stage IV. There are 41 normal tissues, 45 stage I samples, 109 stage II samples, 80 stage III samples, and 37 stage IV samples. For further analysis, all the normal samples were combined with the individual stage. After that, the final samples would be 86, 150, 121, and 78 respectively. These are considered the final four differentially expressed (DE) sets.

For detecting differentially expressed genes (DE), this study mainly used gene expression analysis. To avoid the noises of raw data, data preprocessing is crucial to minimize noise. In addition, performing high-level analysis is a must for quality assessment and preprocessing of sequencing data. Counts Per Million (CPM) value should be calculated for each group of datasets due to filtering lowly expressed genes. Four stages and one normal sample dataset were used for identifying Differentially Expressed Genes. We also considered the TMM (Trimmed Mean of the M-value) algorithm (Robinson and Oshlack, 2010) because it is more effective for comparisons between samples as it does not count gene length or library size.

### Differential gene expression analysis

In this study, the edgeR package (Robinson et al., 2010) in R language was generated from Bioconductor and used to analyze and identify the differentially expressed genes (DEGs) list of the cancerous tissues for four DE sets. These analyses of DEGs were performed between control samples and CRC samples of four different stages. The *p*-values were calculated for all DEGs, which were further adjusted into false discovery rate (FDR) by using the Benjamini–Hochberg method. Fold Change (FC) value for each group was also calculated and only the genes with FDR <0.05 and $\left|logFC\right|\geq 1$ were defined as differentially expressed (DE) genes.

### All DEGs and intersection DEGs

An online tool, Vennplex (Cai et al., 2013), was used to identify not only all DEGs but also the intersection of DEGs between four DEG sets. It also showed the similarities and differences in expression changes between different groups. For this analysis, it is necessary that both gene symbol and $\left|\log2FC\right|$ should be inputted. All common DEGs in these datasets were selected for further study.

### Filtering DEGs for stage-specific network generation

For filtering the remarkable genes, histogram analysis was performed on both clinical and PPI data. The DEGs with logFC > 1.5 and in the PPI network, connections of edge confidence >0.75 were selected as the final DEGs data list as those are mostly connected to colon cancer.

### Stage-specific cluster interaction network generation

In order to identify gene groups that conduct cancer related cellular functions, the functional interaction (FI) network was built. Because one of the motivations of this study is to analyze the functions of the group of genes and the FI network is unable to represent the relations between the coding genes. Four functional interaction networks were constructed using the 4 DE sets (filtered by histogram analysis). The nodes represented the genes, and the edge represented the functional associations and interaction associations between genes. The edge contains the implications of biological function, which provided the basis for the functional analysis. ReactomeFIViz (Wu et al., 2014), a tool in the Cytoscape software (Shannon et al., 2003), was utilized to analyze the functional interactions between all these DEGs.

In this study, the Markov Cluster Algorithm (MCL) was applied to each stage and whether the cluster has five nodes or above were connected, those clusters were selected to analyze the relationship between functional modules for each stage and adjacent stages. Finally, 25 clusters, 33 clusters, 27 clusters, and 29 clusters were chosen for stage I, stage II, stage III, and stage IV respectively. According to the connections of each cluster, seven networks were built, where four networks are for individual stage (stage I, stage II, stage III, and stage IV) and another three is between adjacent stages (between stage I and stage II, between stage II and stage III, between stage III and stage IV).

### Functional evolutionary network generation

The main contribution of this study is the combination of seven networks with four groups (stage I, stage II, stage III, and stage IV) and seven connections (above seven networks). The Cytoscape software was used to make this combined network, called pathway interaction network, in order to analyze the relationship between functional modules. The final graphical view of the pathway interaction network was entitled as the functional evolutionary network. Where the correlation between four DEG sets was obtained and the staged genes involved in the pathway were used for further analysis.

In the functional evolutionary network analysis, the pathway enrichment analysis has been done. It was not necessary to perform a staged biological function analysis because the pathway enrichment provided a variety of the mixed results of pathway. In order to obtain results of functional analysis in stages, this study, therefore, considered using a method that represents pathways by a graph, which are enriched in different stages. This study analyzed two relationships between pathways and between adjacent stages, the first is to determine which pathways were significantly different between stages, and the second determination is to identify which pathways were associated with adjacent stages.

In this study, DAVID (Dennis et al., 2003) was used to perform the Kyoto Encyclopedia of Genes and Genomes (KEGG) analyses in order to identify the biological features of DEGs associated with biological functions as well as elucidate the functional annotation and pathway enrichment analysis to investigate the biological pathways of DEGs in each functional interaction module (criterion: FDR <0.05 and *p*-value <0.05).

### Protein-protein interaction network of significant DEGs

Each biological system within a cell is controlled by proteins. Some proteins do their work on their own, but most of them rely on their interactions with others to perform their biological functions. To understand protein functions and the biological characteristics of the proteins, Protein-Protein Interaction is a must. PPI data became more available through high-throughput technologies and made it possible to visualize PPI data as networks, i.e., PPI networks.

In this study, all filtered DEGs were used to construct the PPI network based on the Search Tool for the Retrieval of Interacting Genes/Proteins (STRING) (Szklarczyk et al., 2018). Here, the species was homo and the score criterion was 0.75. By using cytoHubba, a Cytoscape plug-in, finally selected the hub genes and constructed a network among those candidate genes.

## Results and discussion

### Differentially expressed genes

This table was made with the overlapping samples (323), where various clinical information (patient’s age, gender, different stages information, and vital status) were concerned (Table 1).

**TABLE 1:** Clinical parameters of colon cancer patients.

Group	Subgroup	Frequency	Percent
Age	<60	101	31.2
	> = 60	222	68.7
Gender	Male	173	53.5
	Female	150	46.4
Histology	Adenocarcinoma	282	87.3
	Mucinous adenocarcinoma	41	12.7
Stage	Stage I	49	15.1
	Stage II	132	40.8
	Stage III	87	26.9
	Stage IV	44	13.6
T stage	Tis, T1-3	278	86
	T4	45	13.9
N stage	N0	194	60
	N1+N2	129	39.9
M stage	MX, M0	274	84.8
	M1	44	13.6
Vital status	Alive	244	75.5
	Dead	79	24.4

A total of 312 samples were processed and normalized, where 271 colon cancer samples and 41 normal tissues were present. In the colon cancer samples, there were 45 samples for stage I, 109 for stage II, 80 for stage III, and 37 for stage IV. Finally, the DEGs were identified using adjusted and cut-off criteria. A total of 1,574, 1,607, 1,468, and 1,574 significant DEGs were found between healthy and colon cancer stages I, II, III, and IV, respectively.

### All DEGs and intersection DEGs

**FIGURE 2:**
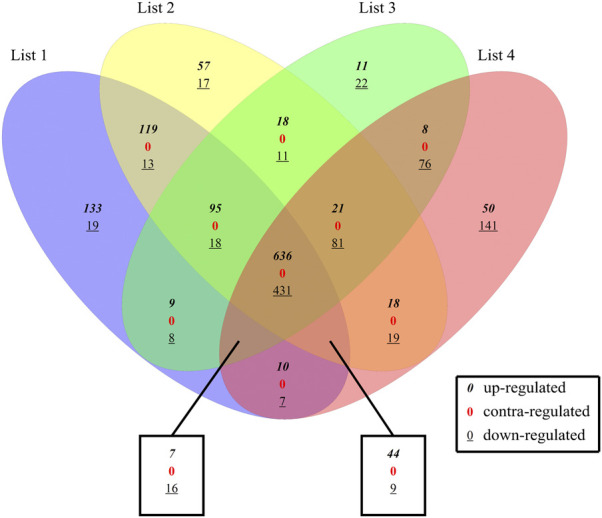
Comparison between 4 DE sets. List1: S1 and Normal, List2: S2 and Normal, List3: S3 and Normal, List4: S4 and Normal; where *0* indicates up-regulated genes, $\underline{0}$ represents down-regulated genes and 0 denotes contra-regulated genes (however there are no contra-regulated genes).

There were a total of 2048 DE genes among 4 DE sets. 1,067 common DEGs were revealed through intersect function, including 1,185 upregulated and 863 downregulated DEGs in comparison with controls. And those common DEGs are defined as intersection DEGs. All the DEGs in the four sets were compared with each other by Vennplex (Figure 2) and it is found that downregulated genes were less compared with upregulated genes in each set. There were no contra-regulated DEGs which suggested that the process (molecular function) of colon cancer at all stages is identical.

### Filtering DEGs for stage-specific network generation

Before the filtering process, there were 1,574, 1,607, 1,468, and 1,574 genes found for stages I to stage IV respectively. After making the histogram analysis based on the PPI network to filter these genes to get significant DE genes the number decreased in Table 2.

**TABLE 2:** Filtered DEGs.

Stage	All DEGs (before filtering)	Filtered DEGs (logFC >1.5 and PPI edge confidence >0.75)
Stage I	1,574	589
Stage II	1,607	569
Stage III	1,468	484
Stage IV	1,574	534

### Stage-specific cluster interaction network generation

**FIGURE 3:**
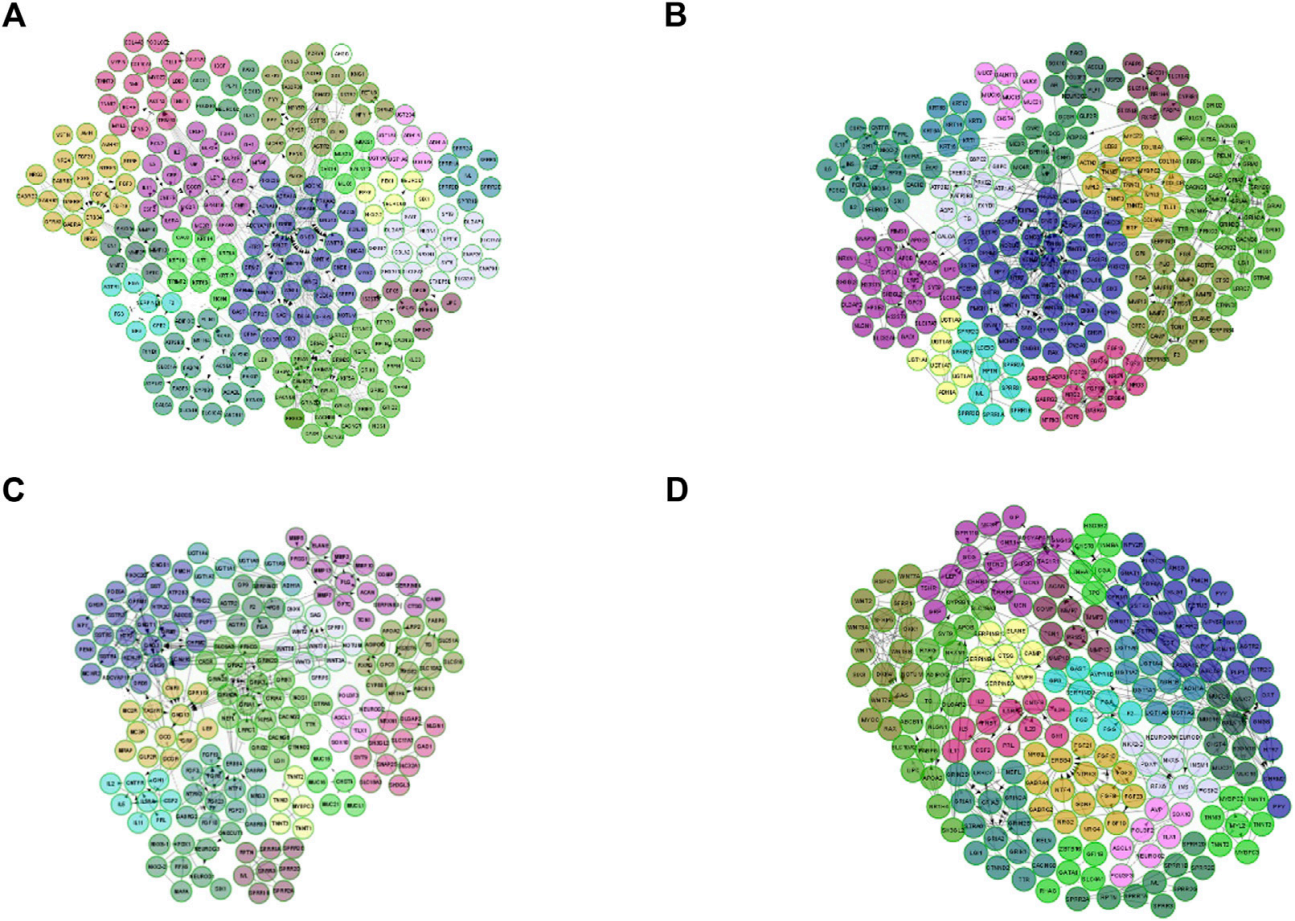
FI network for each stage, where each node is a differentially expressed gene and edge represents the interaction between genes. **(A)** is the FI network for stage I, **(B)** represents the FI network built by DEG-Stage II, **(C)** and **(D)** are the FI network for stage III and stage IV.

According to the DEGs detected in each stage of CRC, we constructed four FI networks at each stage, respectively as the figure shows. Where each node represents a differentially expressed gene. Edge represents the interactions between genes. Figures 3A–D are the FI network constructed by DEG-stage1, DEG-stage2, DEG-stage3, DEG-stage4, respectively. These DEGs normally formed some modules to conduct their cellular functions. That’s why MCL clustering was applied and 17, 15, 16, and 18 modules were found after the clustering at individual stages, respectively. These modules have a higher probability to enrich some functions of colon cancer.

### Cluster interaction networks

Then the cluster interaction networks are constructed for each stage and adjacent stage. There are 7 cluster interaction networks in total. These networks are constructed based on the FI network of each cancer stage by using the MCL cluster algorithm. Where each cluster represents the functions of biological mechanisms and for the investigation of the complex molecular mechanisms of colon cancer these clusters may play important roles. In order to uncover the associations between genes and functions, the interaction between clusters is constructed, where each cluster is treated as a vertex within the interaction and the number of connections between genes in two corresponding clusters as the edge weight. According to the FI network used here, the weights denote the degree of association between clusters. The wider the edges the more closely biology functions between clusters.

### Functional evolutionary network analyses

Examining the functional modules of functional interaction networks at different stages of cancer can provide insight into the functional evolution of the disease and the strength of associations between genes at different stages. This is because these functional modules may contain overlapping genes, and analyzing them can provide valuable information about the progression of the disease.

**FIGURE 4:**
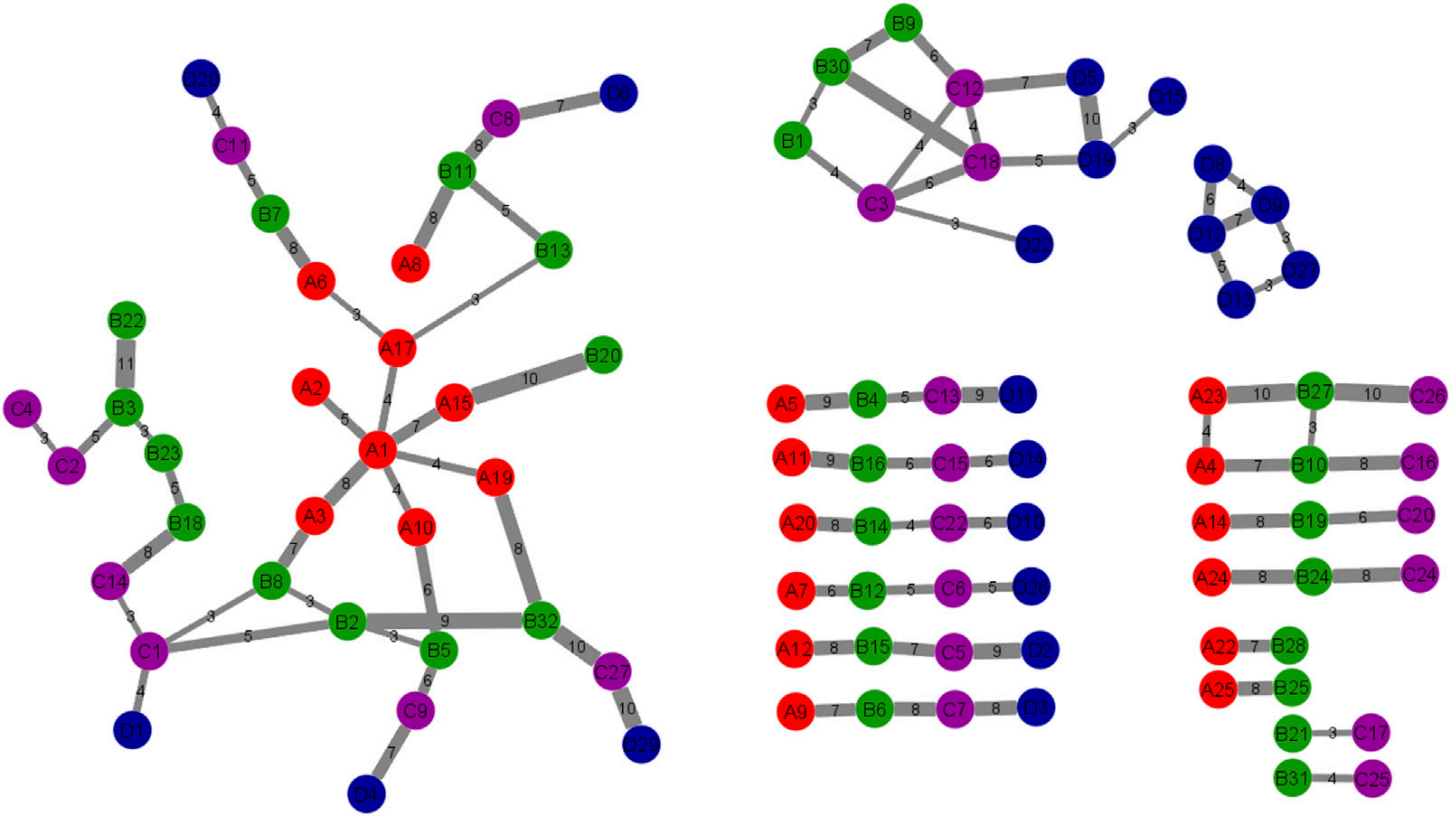
The Pathway Interaction Network. The correlation between 4 DE sets of pathways was obtained and expressed in this network. This network was constructed by combining the 7 cluster networks. Each node is a cluster of different stages which contained several numbers of genes and the edges represent the number of related genes. The thick edges were selected by overlapping genes counts between connected nodes. Red nodes or “A”: DE set I, green nodes or “B”: DE set II, purple nodes or “C”: DE set III, blue nodes or “D”: DE set IV.

Figure 4 shows the pathway interaction network between the four stages. Red nodes or “A”: DE set I, green nodes or “B”: DE set II, purple nodes or “C”: DE set III, blue nodes or “D”: DE set IV. Thick edges were constructed by overlapping gene counts. This pathway interaction network is a major contribution to the field of pathway enrichment analysis. The network was constructed after doing many analyses to get the significant pathways. This network is the result of combining 7 cluster networks. There are four connected networks and some other pathways among the four stages. In this study, the large network is more significant because all the stages are connected in different ways and the other three are connected to one, two, or three stages. This network is the final functional evolutionary network. In terms of the connections between functional modules between adjacent stages, this study constructs this functional evolution network. Nodes indicate the modules. Edges reflect the connections between modules at colon cancer stages. The overlapping genes between functional clusters at adjacent stages are represented by the network’s edges. The more overlapping genes there are between adjacent stages of functional modules, the thicker the edges. In the following sections, this network will be analyzed to investigate the molecular mechanism of colon cancer.

### Pathway enrichment between edges of adjacent cluster

In this study, by analyzing the pathway interaction network and doing KEGG pathway enrichment analysis, 15 common pathways were found. They are: Neuroactive ligand-receptor interaction, Glutamatergic synapse, Circadian entrainment, Nicotine addiction, Retrograde endocannabinoid signaling, cAMP signaling pathway, Dopaminergic synapse, Adrenergic signaling in cardiomyocytes, Amphetamine addiction, Bile secretion, Wnt signaling pathway, Dilated cardiomyopathy, Long-term potentiation, Cardiac muscle contraction, Melanogenesis. Among them, cAMP signaling pathway controls essential physiological activities such as metabolism, secretion, calcium homeostasis, muscular contraction, cell destiny, and gene transcription (Cho-Chung et al., 2002). The neuroactive ligand-receptor interaction pathway is a group of receptors and ligands on the plasma membrane that are linked to intracellular and extracellular signaling pathways and this pathway is associated with prostate cancer (He et al., 2018). The APC mutant colon cancer cells remain dependent on Wnt and suppress the production of secreted Wnt antagonists epigenetically (He et al., 2005). Ke yang et al. demonstrated that the IWP inhibitors inhibit the WNT signaling pathway in colon cancer cells by disrupting the WNT ligand (Yang et al., 2016).

### Pathway enrichment between edges of all stages

For analyzing the different pathways, KEGG pathway analysis was done and about five significantly different pathways were found. Pathways in cancer, Serotonergic synapse, Bile secretion, Hypertrophic cardiomyopathy (HCM), and Dilated cardiomyopathy are significantly different at all stages. The findings pathway namely Serotonergic synapse pathway is a neurotransmitter which is widely distributed in the vertebrate central nervous system, and it serves as a target for many physiologic regulations like modulators of gene transcription, steroids and neurotrophic factors. Ionotropic GluRs and 5-HT3 receptors mediate rapid synaptic transmission *via* serotonergic fibers making direct synaptic connections with GABAergic neurons (Maejima et al., 2013). There are active transport systems within hepatocytes and cholangiocytes. Hepatocytes secret their bile by secreting conjugate bilirubin, bile salts, cholesterol, phospholipids, and water into their canaliculi (Hundt et al., 2022). A primary myocardial disorder associated with the autosomal dominant pattern of inheritance is hypertrophic cardiomyopathy (HCM), which can be distinguished from other cardiovascular disorders primarily by the presence of myocyte hypertrophy, fibrillar disarrays, and interstitial fibrosis as histological characteristics. Dilated cardiomyopathy (DCM) is a heart muscle illness described by dilation and impaired contraction of the left or both ventricles that is responsible for progressive heart failure and sudden cardiac death from ventricular arrhythmia.

### Heatmap analysis for pathways

To identify the significant pathways, heatmap analysis was done based on the *p*-values of each stage. Table 3 shows a total of 15 pathways with the *p*-values of each stage.

**TABLE 3:** Pathways table.

Pathways	S1 *p*-value	S2 *p*-value	S3 *p*-value	S4 *p*-value
Neuroactive ligand-receptor interaction	2.80E-19	2.60E-11	2.90E-13	1.50E-02
Glutamatergic synapse	5.40E-14	1.40E-05	2.20E-05	3.30E-04
Circadian entrainment	4.50E-10	3.20E-06	5.40E-05	1.40E-04
Nicotine addiction	3.90E-09	4.80E-11	1.90E-10	5.30E-08
Retrograde endocannabinoid signaling	1.00E-08	5.30E-06	6.20E-04	1.70E-02
cAMP signaling pathway	3.60E-07	1.30E-06	7.80E-07	5.80E-04
Dopaminergic synapse	1.40E-06	3.70E-05	2.10E-03	5.60E-04
Adrenergic signaling in cardiomyocytes	3.40E-06	6.60E-05	5.60E-04	7.90E-04
Amphetamine addiction	4.60E-05	1.80E-06	5.90E-05	2.40E-05
Bile secretion	6.40E-05	2.70E-05	7.30E-04	5.90E-03
Wnt signaling pathway	9.30E-05	1.00E-05	5.60E-04	5.10E-07
Dilated cardiomyopathy	2.60E-04	1.10E-06	2.20E-06	4.80E-06
Long-term potentiation	3.30E-04	1.90E-04	4.90E-03	4.00E-04
Cardiac muscle contraction	7.20E-04	4.80E-06	1.20E-05	4.50E-05
Melanogenesis	8.40E-04	2.90E-04	2.00E-02	1.90E-03

**FIGURE 5:**
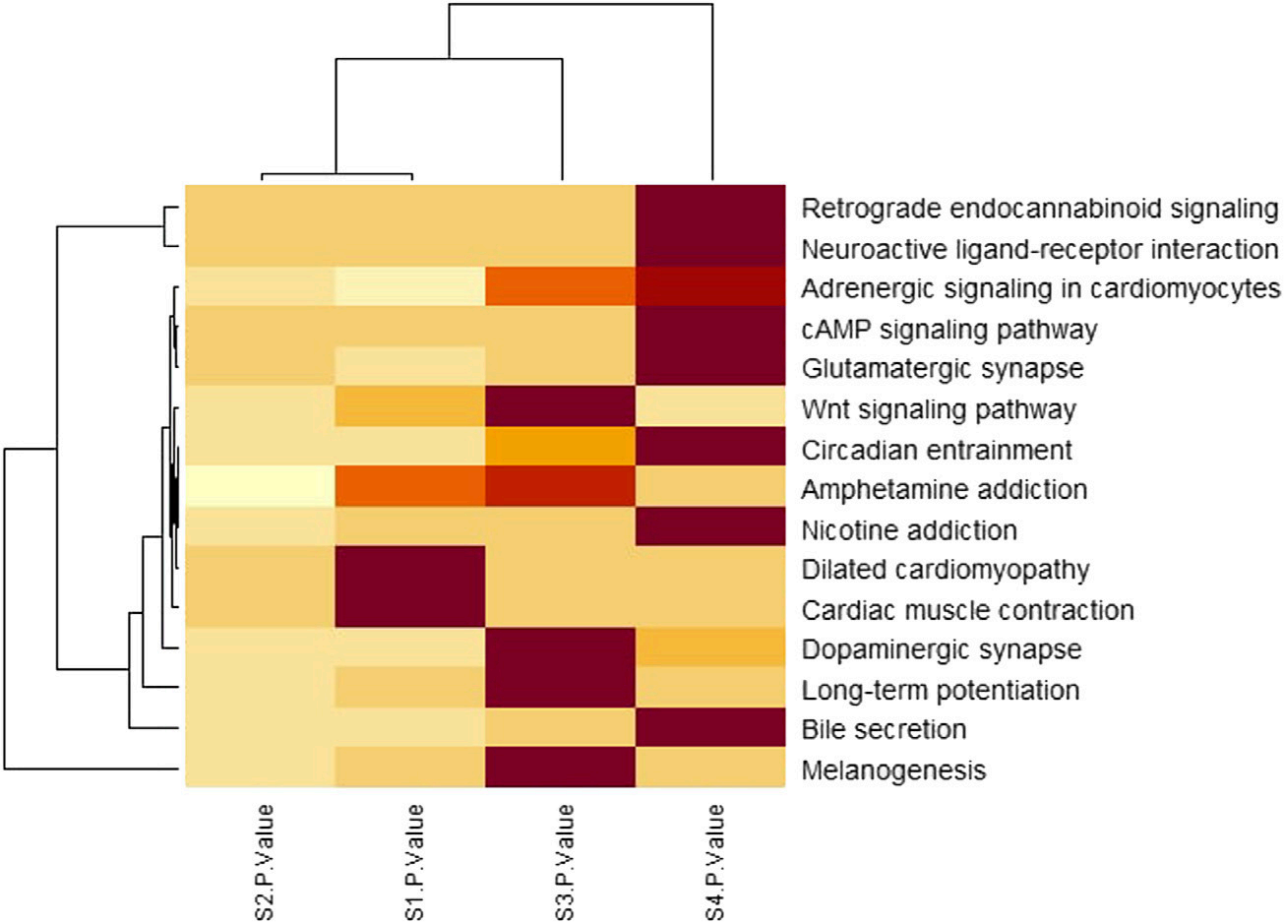
Pathway analysis by clustered heatmap analysis of four stages where pathways were selected by the KEGG pathway enrichment analysis. The functional components identified from the KEGG pathway enrichment analysis are presented row-wise (right side) and each stage is presented column-wise (bottom). The color intensities indicate the enrichment score of each KEGG pathway with a color gradient that moves from light yellow to maroon. The dendrogram indicates the similarity between pathways as well as stages.

To investigate the significant biological functions of DEGs, heatmap analysis was done on each stage (Figure 5) and among those pathways, some are significantly different such as neuroactive ligand-receptor interaction, Glutamatergic synapse, Circadian entrainment, and Nicotine addiction have low *p*-value (from Table 3) and a large number of genes.

From Table 3; Figure 5, it is clear that the *p*-values of neuroactive ligand-receptor interaction, Glutamatergic synapse, Circadian entrainment, and Nicotine addiction change and increase from the initial stage to later stage and Figure 5 also shows that the color changes from light yellow to marron and it is said for heatmap analysis is that bright color indicates high activity and dark color is *vice versa*.

Fang et al. and Liu et al. has shown that the neuroactive ligand-receptor interaction signaling pathway is linked to bladder cancer and renal cell carcinoma progression (Fang et al., 2013; Liu et al., 2015). It is believed that glutamatergic synapse pathways play a crucial role in a large variety of normal physiological functions due to their links to many other neurotransmitter pathways and neurodevelopmental disorders and injuries are strongly associated with glutamate dysfunction (Glutamatergic Synapse Pathway, 2022). Circadian entrainment includes retinal sensitivity as well as circadian variations in the retina that contribute to the regulation of retinal diseases and these circadian disorders are related to entrainment deficits (Golombek and Rosenstein, 2010). It is shown in various studies that Nicotine addiction increased approximately 50% chances of colon cancer because smoking has been associated with adenomatous polyps (Slattery et al., 1997). The genes encoding these pathways may be important in the molecular development of these pathways since they are strongly associated with function.

### PPI network of significant DEGs

**FIGURE 6:**
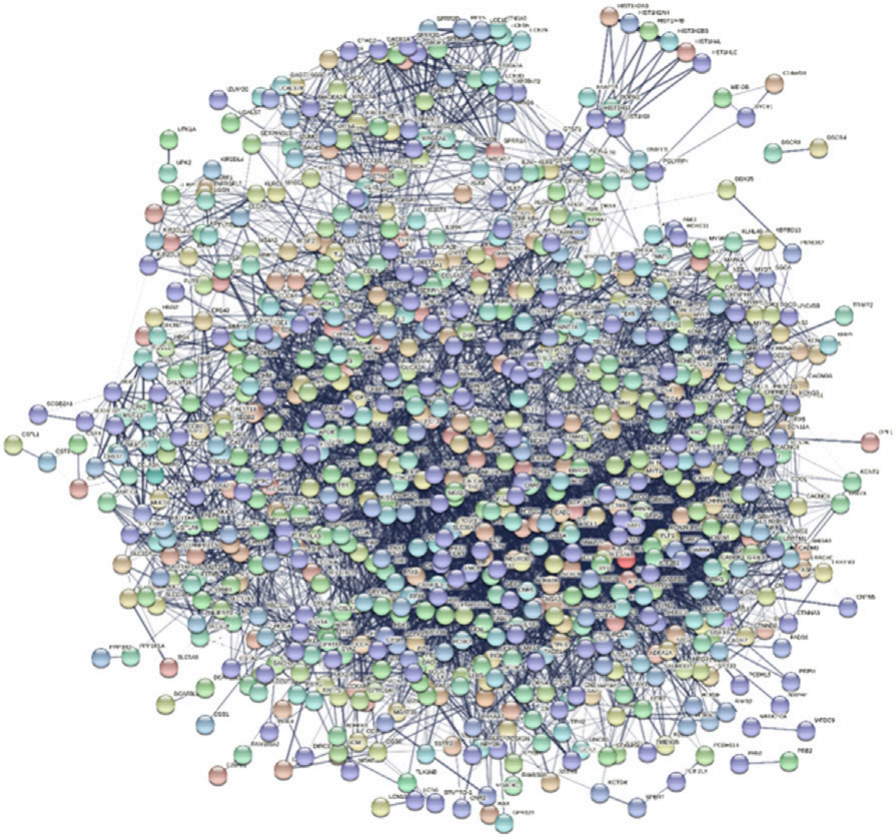
Protein-Protein Interaction (PPI) Network of intersection DEGs.

A Protein-Protein Interaction network (PPI) is a visual framework for better understanding protein functional organization. When the iteration between genes was found by above analysis, the PPI network was also constructed to check their relationship because the PPI network is the widely used network to see whether there is a strong relationship or not. In the PPI network, all the significant DE genes were investigated and a PPI network was built. Among these DEGs, some DEGs also appeared in functional interaction network and some DEGs are also known colon cancer related genes, such as CXCL11, ADH1B, PYY, SLC17A7, and so on. The network involved 794 nodes, and 7,153 edges (Figure 6).

### Identifying stage related hub genes

**FIGURE 7:**
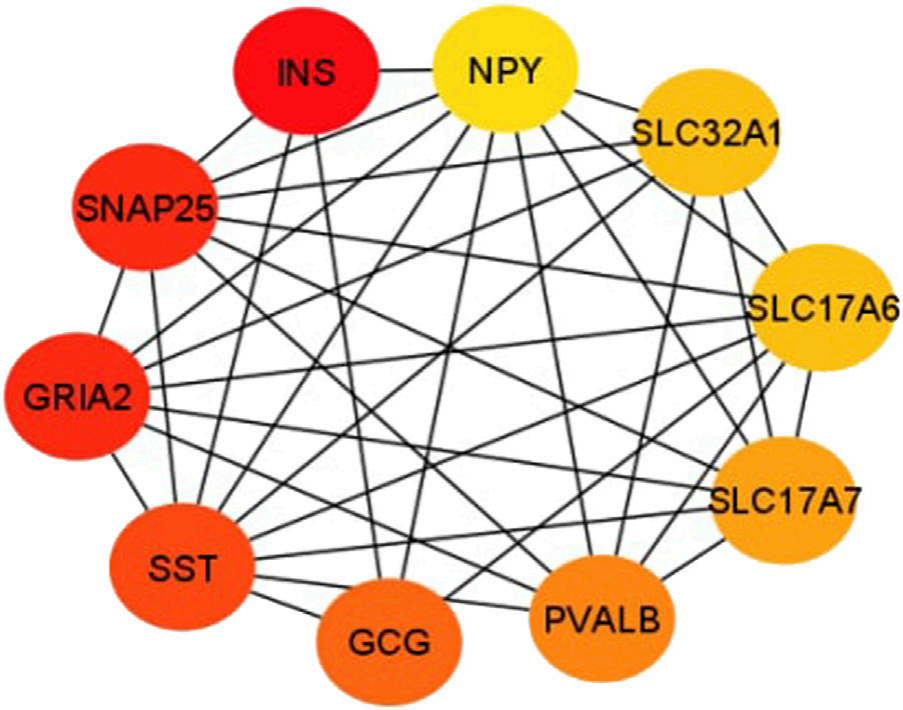
This sub-network shows the connection between hub genes. Red color denotes the degree of connectivity is highest and yellow color means the lowest degree of connectivity and the color changes from red to yellow means the degree of connectivity decreases.

In this study, the top 10 hub genes (INS, SNAP25, GRIA2, SST, GCG, PVALB, SLC17A7, SLC32A1, SLC17A6, and NPY) were identified according to the degree of connectivity of the DEGs and arranged it in a descending order to find the highest degree of connectivity (Table 4). Figure 7 showed the network between the top 10 hub genes.

**TABLE 4:** Top 10 hub genes with higher degree of connectivity and betweenness value.

Gene	Degree of connectivity	Betweenness value
INS	147	82257.16327
SNAP25	97	10475.22985
GRIA2	96	7915.71159
SST	90	11508.22796
GCG	89	12209.61485
PVALB	85	17121.22796
SLC17A7	84	7167.58773
SLC32A1	83	5748.84911
SLC17A6	83	8895.32674
NPY	81	9424.62421

## Conclusion

Gene expression profiling of four CRC stages and healthy colorectal tissue was investigated in this study to learn more about colon cancer mechanisms and stage-related genes of colon cancer. After selecting the DEGs for four stages FI network was constructed and an MCL graph clustering algorithm was performed on the FI network to extract some modules of colon cancer. Then we perform the cluster interaction network to get more specific and significant biological functions. After that, pathway enrichment analysis was done with the MCL modules and in order to improve the analysis, a functional evolutionary network was constructed which described the relationships among pathways at each stage. Finally, the PPI network was constructed using the strong common genes among 4 DE sets. Based on the degree of connectivity, 10 hub genes were chosen as the potential colon cancer stage-related genes and those are INS, SNAP25, GRIA2, SST, GCG, PVALB, SLC17A7, SLC32A1, SLC17A6, and NPY.

Comparing the relationships between the same pathways and different pathways in neighboring DE sets was a very useful way to analyze the staged biological functions of colon cancer. KEGG pathway enrichment analysis at adjacent stages showed that Pathways in cancer, Serotonergic synapse, Bile secretion, Hypertrophic cardiomyopathy (HCM), and Dilated cardiomyopathy are significantly different at all stages. And neuroactive ligand-receptor interaction, Glutamatergic synapse, Circadian entrainment, and Nicotine addiction are the significant pathways among each stage. Overall, this study has identified novel candidate biomarkers and pathways associated with colon cancer.

In conclusion, 10 potential colon cancer stage-related genes, four significant pathways, and some biological information were discovered on the disease in this study. These results might provide some significant information about the stages and the genes would serve as staged biomarkers of colon cancer. Though the current findings provided valuable information for early detection and prevention, as well as a viable therapeutic target for CRC. However, there are certain limitations to the research: i) No mRNAs or miRNAs were used with TCGA. ii) In order to verify the accuracy of the results, some biological experiments will still be necessary.

## Data Availability

The original contributions presented in the study are included in the article/Supplementary Material, further inquiries can be directed to the corresponding author.
